# Complement in HER2-Positive Breast Cancer: A Conditionally Protective Mechanism Under Attack

**DOI:** 10.3390/antib15050091

**Published:** 2026-10-05

**Authors:** Dobromira Petkova, Ivaila Kuleva, Zhasmin Stoyanova, Boryana Ilcheva, Albena Fakirova, Zhasmina Mihaylova, Maria Radanova

**Affiliations:** 1 Clinic of Medical Oncology, Military Medical Academy, 1000 Sofia, Bulgaria; dmpetkovva@abv.bg (D.P.); zhasmina.mihaylova@gmail.com (Z.M.); 2 Department of Biology, Medical University of Varna, 9000 Varna, Bulgaria; ivayla.kuleva@mu-varna.bg; 3 Department of Biochemistry, Molecular Medicine and Nutrigenomics, Medical University of Varna, 9000 Varna, Bulgaria; jasmin042004@gmail.com; 4 Clinic of Pathology, Military Medical Academy, 1000 Sofia, Bulgaria; bukiu@abv.bg (B.I.); afakirova@gmail.com (A.F.)

**Keywords:** complement, HER2+ breast cancer, membrane-bound complement regulatory proteins, mCRP, trastuzumab, pertuzumab, zanidatamab

## Abstract

The therapeutic efficacy of anti-HER2 monoclonal antibodies in breast cancer has traditionally been attributed to direct inhibition of signaling and antibody-dependent cellular cytotoxicity (ADCC). Recent evidence, however, has established complement activation through the classical pathway as a possible adjunct mechanism underlying the synergistic activity of the trastuzumab and pertuzumab combination. This synergy is mediated by complement-dependent cytotoxicity (CDC) and complement-dependent cellular phagocytosis (CDCP), and is lost upon C1q deletion in preclinical models. Tumor cells evade complement-mediated killing through overexpression of membrane-bound complement regulatory proteins (mCRPs), particularly CD55 and CD59, which correlate with shorter disease-free survival in patients treated with trastuzumab. C1q itself plays a dual, context-dependent role: it acts as a complement-independent tumor suppressor in HER2-positive breast cancer while promoting tumor progression in most other malignancies. Emerging therapeutic strategies, including biparatopic antibodies such as zanidatamab, complement-redirecting immunoconjugates, and mCRP-targeting approaches, aim to overcome complement resistance and enhance antitumor immunity. On this basis, we propose that HER2-positive breast cancer represents a conditionally complement-protective tumor, in which the balance between antibody-directed complement activation and mCRP-mediated evasion determines treatment outcome. This review evaluates the current evidence linking complement to anti-HER2 therapy and highlights future directions for successful treatment.

## 1. Introduction

Breast cancer is a major cancer in women, accounting for about a quarter of all cancer cases worldwide [1]. Nonetheless, mortality from the disease has decreased and survival has improved across all demographic groups. This positive trend is mainly due to new prognostic and diagnostic methods, as well as the development of effective, targeted and personalized therapies [2,3].

The HER2+ molecular subtype of breast cancer is characterised by *ERBB2* gene amplification and overexpression of the human epidermal growth factor receptor 2 (HER2) protein, which represents 15–20% of invasive breast cancer cases. This subtype is associated with aggressive clinical behaviour, increased metastatic potential, and poor prognosis [4,5]. Despite the poor prognosis of the HER2+ molecular subtype, the introduction of monoclonal antibodies targeting HER2 has reduced the risk of mortality by more than 30%, and five-year overall survival for early-stage disease exceeds 90% [6]. Still, about 25% of patients relapse within 10 years, indicating the need for a better understanding of the mechanisms underlying therapy success and the emergence of resistance [6].

HER2 is a transmembrane tyrosine kinase constitutively active receptor in the EGFR/ErbB family, which does not require ligand binding for activation, and it is known as a potent driver of oncogenesis [7]. The development of HER2-targeted monoclonal antibodies (mAbs) has transformed therapy for HER2-positive breast cancer. Trastuzumab, a humanized IgG1 mAb targeting the extracellular domain IV (ECD4) of HER2, was the first anti-HER2 therapy and remains central to the treatment of both early-stage and metastatic disease [5]. Pertuzumab, another humanised IgG1 antibody, binds a separate epitope on extracellular domain II (ECD2) and inhibits ligand-dependent HER2 heterodimerization with other ErbB family members, especially HER3 [8]. The combination of trastuzumab, pertuzumab and docetaxel is now the standard first-line treatment for HER2+ metastatic breast cancer. The CLEOPATRA trial demonstrated significant improvements in both progression-free survival and overall survival with this therapy [9,10].

In the neoadjuvant setting, dual HER2 blockade with trastuzumab and pertuzumab in combination with chemotherapy achieves higher rates of complete pathological response and a better prognosis than trastuzumab monotherapy, as demonstrated by the NeoSphere trial [11]. It is therefore established as the standard of care in the (neo)adjuvant setting [12] and is the current European Society for Medical Oncology (ESMO) clinical practice guideline [13].

Trastuzumab and pertuzumab block HER2-driven signaling, prevent receptor dimerization, and promote receptor internalization [7,8]. Their clinical efficacy largely comes from immune effector functions mediated by the IgG1 Fc region, including antibody-dependent cellular cytotoxicity (ADCC) via Fcγ receptor engagement on NK cells and macrophages [14] and antibody-dependent cellular phagocytosis (ADCP) [15]. A third Fc-dependent mechanism for complement-dependent cytotoxicity (CDC) has received less attention in anti-HER2 therapy, but recent data suggest it may be more relevant than previously thought. Moreover, complement is now recognised as a context-dependent modulator of tumor biology, with both its canonical and non-canonical functions affecting cancer progression [16,17].

Initial in vitro studies showed that trastuzumab alone induced minimal CDC against HER2-overexpressing cells [18,19]. Subsequent work demonstrated in preclinical models that the combination trastuzumab–pertuzumab activated the classical complement pathway and that this activation contributes to its antitumor efficacy [15]. The clinical relevance of complement is further supported by the observation that overexpression of membrane-bound complement regulatory proteins (mCRPs), particularly CD55 and CD59, on tumor cells is associated with shorter disease-free survival in HER2+ patients treated with trastuzumab [20]. By protecting tumor cells from complement-mediated lysis, these regulators are a major cause of resistance to CDC [19,21]. Several strategies are currently being developed to overcome this resistance [18,19,22,23].

C1q plays two roles in this context. It is the molecule that recognizes antibodies on the tumor cell and initiates the classical pathway of complement activation [24], and it also suppresses HER2-induced tumor growth through mechanisms outside the complement cascade [25]. Its presence therefore determines both whether anti-HER2 antibodies can engage complement and how the tumor behaves in the absence of therapy.

Complement activation by anti-HER2 antibodies, mCRP-mediated resistance, and the role of C1q in the tumor microenvironment have been studied separately, but such a detailed integration of their role has not been made in the literature. We review the current evidence linking complement to the therapeutic efficacy of anti-HER2 antibodies, present the role of mCRPs as determinants of treatment resistance, and highlight emerging strategies to enhance complement-mediated antitumor immunity in HER2+ breast cancer. Based on this evidence, we propose that HER2-positive breast cancer should be considered a complement-protective tumor [16], in which the balance between complement activation by therapeutic antibodies and complement evasion by mCRPs determines treatment outcome.

This article is a narrative review based on the literature gathered from PubMed, Scopus, and Web of Science up to September 2026, using the following search terms: C1q, C3, C8A, CD55, CD59, and CD46, in conjunction with HER2-positive breast cancer, trastuzumab, pertuzumab, zanidatamab, and antibody–drug conjugates. The review includes original experimental studies, clinical cohorts, and clinical trials reporting primary data on complement activation, complement regulation, or complement-related biomarkers in HER2-positive breast cancer or directly relevant models. Review articles were used to provide context, but the conclusions of the present review are based on primary sources.

## 2. Complement Activation by Anti-HER2 Antibodies

### 2.1. General Principles of Complement Activation by Antibodies

Classical complement activation begins with the formation of the C1 complex, including C1q, which recognizes immune complexes with IgG or IgM on target surfaces, and a heterotetramer of serine proteases C1r_2_C1s_2_ [24]. Efficient binding of C1q requires the assembly of six IgG molecules into hexameric Fc platforms on the cell surface via noncovalent Fc:Fc interactions, thereby providing the necessary avidity to initiate the proteolytic cascade [24,26]. The binding of C1q triggers the autoactivation of C1r, which then cleaves and activates C1s. Activated C1s sequentially cleave C4 and C2, generating the C3 convertase (C4bC2b). Cleavage of C3 produces C3b, the key opsonin, and the anaphylatoxin C3a. Accumulation of C3b leads to the formation of C5 convertase (C4bC2bC3b), which cleaves C5 to C5a, a potent anaphylatoxin and chemoattractant for immune effector cells, and C5b, which initiates the assembly of the membrane attack complex (MAC, C5b–9) for target cell lysis, a process known as CDC [27]. Under physiological conditions, this process is tightly controlled by mCRPs—CD55, CD46, and CD59—that inhibit the cascade at different levels, from convertase formation to MAC assembly [27,28,29,30].

The efficiency of classical complement activation depends on multiple factors: IgG subclass, accessibility and membrane proximity of epitopes; antigen density on the cell surface; and the ability of bound antibodies to form hexameric Fc platforms [31,32]. Among the human IgG subclasses, IgG1 and IgG3 are the most effective complement activators [32]. Both trastuzumab and pertuzumab belong to the IgG1 subclass and therefore have the structural prerequisites for good interaction with C1q [18]. However, the presence of an IgG1 Fc domain alone does not guarantee effective complement activation, as the properties of the target antigen and the geometry of the antibody-binding site also play important roles [31]. Zolbetuximab illustrates this. It is a chimeric IgG1 against claudin-18.2 and it mediates efficient CDC against target cells in human serum with the amplitude of lysis correlating directly with cell surface antigen levels [33]; by contrast, trastuzumab, also an IgG1 with an unmodified Fc, produces little or no lysis under comparable conditions (Section 2.2). The difference does not lie in the antibody class but in the target: epitope position, antigen density and the resulting spacing of bound Fc domains determine whether C1q can be bound with sufficient avidity.

### 2.2. Determinants of Complement Activation by Anti-HER2 Antibodies

In vitro studies consistently show that trastuzumab alone induces little or no CDC activity against HER2-overexpressing tumor cells [15,18,19,34]. Several explanations are possible. HER2-bound trastuzumab undergoes rapid receptor-mediated internalization, which reduces surface Fc density [7]. In addition, overexpression of mCRPs, particularly CD55 and CD59, on HER2-positive tumor cells inhibits the complement cascade [19,20].

A third explanation concerns the geometry of antibody binding. Efficient complement activation requires the assembly of bound IgG molecules into hexameric Fc platforms on the cell surface [26,31]. The epitope targeted by trastuzumab (ECD4), together with the density and orientation of antibody binding, may not favor the Fc:Fc interactions needed for hexamerisation, resulting in inefficient C1q recruitment. In support of the latter is the explanation of Mamidi et al. (2013), who suggested that only the combination of trastuzumab and pertuzumab, binding to distinct epitopes of the HER2 extracellular domain, achieves a sufficient number of cell-bound antibodies at the critical distance required for efficient C1q binding and complement activation [18]. Early experimental evidence in this regard was provided by Spiridon et al. (2002), who showed that a mixture of three anti-HER2 monoclonal antibodies fixed complement in a dose-dependent manner and induced approximately two-fold greater lysis of BT-474 breast cancer cells compared to each of them alone [35]. The same principle has been demonstrated in studies of combinations of antibodies targeting the folate receptor and EGFR, which were found to require non-overlapping epitopes to induce CDC [36,37]. Mamidi et al. (2013) confirmed that the trastuzumab–pertuzumab combination deposits complement on HER2+ tumor cells, but effective CDC required simultaneous neutralization of the membrane regulators CD46, CD55, and CD59 [18]. About a decade later, Tsao et al. (2022) demonstrated in vivo that the combination of trastuzumab with pertuzumab induced potent activation of the classical complement pathway in HER2+ tumors, implicating complement as a mechanism of their therapeutic synergy [15].

The authors provide the following evidence that the synergistic activity of the antibody combination is mediated by CDC rather than by enhanced ADCC, ADCP, or by inhibition of HER2 signaling: (1) the synergistic antitumor effect is maintained in SCID-beige mice, which lack adaptive immunity and NK cells; (2) in vitro complement activation requires both antibodies and is eliminated by treatment with a C1 inhibitor or by heat inactivation of serum complement; and (3) in vivo genetic deletion of C1qa abolishes the synergistic therapeutic benefit of combining trastuzumab with pertuzumab compared with trastuzumab monotherapy, whereas pharmacological inhibition of C1 significantly reduces the efficacy of the antibody combination [15]. Two downstream effector mechanisms of complement have been identified: direct lysis by MAC formation–CDC and enhanced macrophage-mediated phagocytosis following C3b/iC3b deposition–complement-dependent cellular phagocytosis (CDCP) [15].

Taken together, these results indicate that the two anti-HER2 antibodies, binding different epitopes, are sufficient to activate the classical complement pathway, which is not possible with either antibody alone. However, the cytotoxicity observed is condition-dependent: the combination of antibodies readily opsonizes tumor cells with C3b in human serum [15,18] and mediates lysis both in vivo and in vitro with rabbit complement [15], whereas in human serum direct lysis remains minimal [18,22], due to the limiting effect of mCRP.

**Figure 1:**
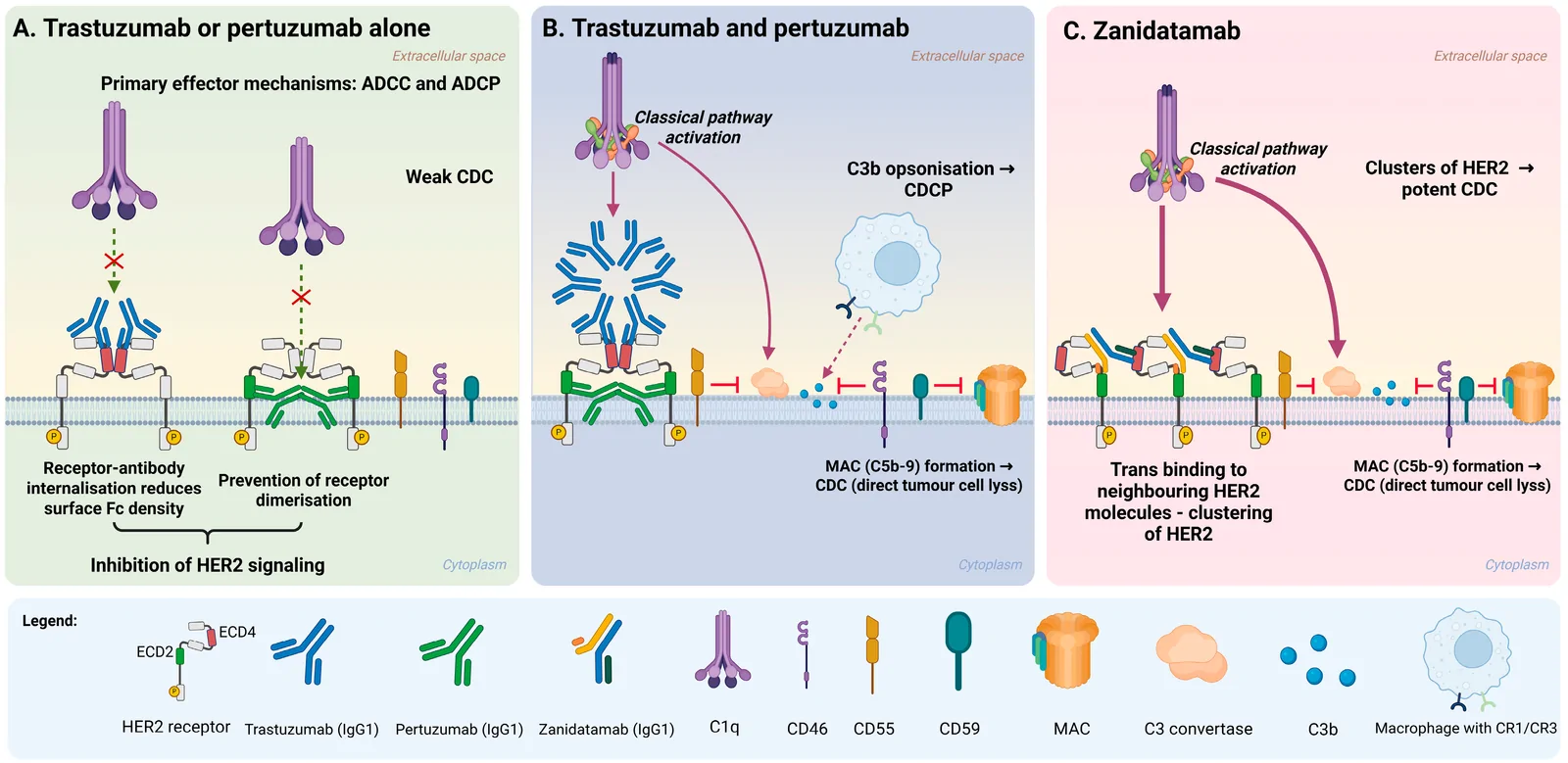
Complement activation by anti-HER2 antibodies. (**A**) Trastuzumab or pertuzumab alone. Trastuzumab or pertuzumab as single agents exert antitumor effects primarily through ADCC and ADCP. Neither antibody alone achieves sufficient Fc density on the tumor cell surface for efficient binding to C1q and activation of the classical pathway, resulting in poor CDC. Receptor-antibody internalization further reduces surface Fc density (trastuzumab), whereas pertuzumab primarily prevents receptor dimerization. (**B**) Trastuzumab and pertuzumab. The combination of trastuzumab (binding to ECD4) and pertuzumab (binding to ECD2) on the same HER2 molecule increases surface Fc density, allows binding of C1q, formation of the C1 complex, and activation of the classical pathway. This results in two effector mechanisms: formation of MAC (C5b-9), leading to direct tumor cell lysis (CDC), and opsonization of C3b, leading to macrophage-mediated phagocytosis via CR1/CR3 (CDCP). mCRPs (CD55, CD46, CD59) are present on the surface of tumor cells but are insufficient to block complement activation by the T + P combination. (**C**) Zanidatamab. Zanidatamab, a biparatopic anti-HER2 antibody, binds to ECD2 and ECD4 on adjacent HER2 molecules in a trans configuration, inducing unique HER2 clustering, leading to potent activation of the classical pathway and CDC. Note: ADCC and ADCP also contribute to the overall efficacy of anti-HER2 antibodies but are not shown, as the figure focuses on complement-mediated mechanisms.

Interestingly, the geometric effect that is achieved with two separate antibodies can be achieved by a single biparatopic molecule. Zanidatamab binds two non-overlapping HER2 epitopes on adjacent receptor molecules in trans, inducing HER2 clustering and potent CDC in the presence of human serum (Figure 1) [22].

The requirement for two epitopes can be applied to the other HER2-directed agents now in use. Antibody–drug conjugates such as T-DM1 and T-DXd, constructed on trastuzumab or on an IgG1 carrying its binding sequence [38,39], retain an unmodified Fc, so the limitation is not in the Fc but in the single epitope, which restricts hexamer assembly. Rapid internalisation acts in the same direction, and ADC design maximises it. The characterisation studies of T-DM1 and T-DXd report HER2 signalling blockade and inhibition of ectodomain shedding and ADCC, but do not assess complement [39,40,41]. A recent review nevertheless lists CDC among the Fc-mediated functions of these conjugates [42]. ADCs probably inherit the complement behaviour of the antibody on which they are built, and we therefore expect that an ADC also built on a biparatopic backbone would retain CDC, since epitope geometry largely determines complement activation.

Receptor density sets a further limit. HER2 density in HER2-low and HER2-ultralow tumors is roughly an order of magnitude lower than in HER2-positive tumors [43], so complement activation is unlikely there even when two epitopes are engaged. NSABP B-47 is consistent with this, as adding trastuzumab to adjuvant chemotherapy gave no benefit in patients with HER2-low tumors [44]. T-DXd is effective in both HER2-low and HER2-ultralow disease [45,46], but this efficacy fails both conditions at once and cannot be complement-dependent. The mechanism discussed in this review is therefore confined to HER2-positive disease.

## 3. Membrane-Bound Complement Regulatory Proteins as Determinants of Resistance to Anti-HER2 Therapy

### 3.1. Mechanisms of Complement Evasion by Membrane-Bound Complement Regulatory Proteins

Membrane-bound complement regulatory proteins (mCRPs) protect host cells from bystander complement-mediated damage [27,29]. Three mCRPs are especially relevant in anti-HER2 therapy: CD55 (decay-accelerating factor, DAF), CD59, and CD46 (membrane cofactor protein, MCP). Each inhibits the complement cascade at a different stage. CD55 accelerates the decay of C3 and C5 convertases (C4bC2b and C4bC2bC3b), preventing cascade amplification [29]. CD46 acts as a cofactor for factor I-mediated cleavage of C3b and C4b, promoting their inactivation. CD59 binds to C5b-8 and prevents C9 incorporation into the MAC, blocking cell lysis [29,30].

CD55, CD59 and CD46 are frequently overexpressed in breast, colorectal, ovarian and lung carcinomas, where their combined activity shields tumor cells from complement-mediated lysis and limits the efficacy of complement-activating monoclonal antibodies [16,17,21,47].

### 3.2. Clinical Significance of mCRP Expression in Breast Cancer: Context-Dependent Effects

The prognostic significance of mCRP expression in breast cancer depends on the treatment context of the disease. In large, unselected cohorts of breast cancer patients, mostly untreated with anti-HER2 monoclonal antibodies, Madjd et al. (2003, 2004) showed that the loss of CD55 and CD59 was associated with a higher histological grade, poorer prognostic indices, and shorter survival (Table 1) [48,49]. These disconcerting findings suggest that, in the absence of antibody therapy, mCRP may influence tumor biology through functions unrelated to complement regulation, including CD55-mediated regulation of cell adhesion and migration through its interaction with CD97 [50,51], and the involvement of CD59 in intracellular signaling pathways affecting tumor differentiation [48,52].

However, when treatment included complement-activating anti-HER2 antibodies, the prognostic impact of mCRP expression was reversed. Liu et al. (2014) showed that high expression of CD55 or CD59 in HER2+ patients on adjuvant trastuzumab was associated with more relapses and shorter disease-free survival, whereas CD46 had no prognostic significance (Table 1) [20].

The same reversal appeared at the population level: high CD55 and CD59 expression was inversely associated with relapse-free survival (RFS) in HER2-positive breast cancer, while high *C1QA* gene expression was associated with better overall survival (OS) [15]. Ouyang et al. (2016) provided further evidence that CD59 overexpression in a cohort of breast cancer patients is associated with a worse prognosis and that CD59 knockdown inhibits tumor cell growth both in vitro and in vivo (Table 1) [53]. All these clinical, bioinformatic and experimental data from different studies indicate that tumor expression of mCRP contributes to resistance to anti-HER2 therapy and has opposite prognostic roles with and without therapy. Therefore, the treatment context must be taken into account when interpreting mCRP as a biomarker. These markers are measured in tissue and have no circulating equivalent. Soluble CD55 and CD59 released from the GPI anchor inhibit complement about two hundred times less efficiently than the membrane-bound protein [54], and their relationship to tumor expression remains unknown.

**Table 1:** Clinical and preclinical evidence for the role of membrane-bound complement regulatory proteins (mCRPs) in HER2+ breast cancer.

mCRP(s)	Treatment Context	Study Type/Model	Key Finding	References
Clinical evidence				
CD55 CD59 CD46	During anti-HER2 therapy	Retrospective clinical cohort; *n* = 65 HER2+ BC patients; adjuvant trastuzumab	High CD55 (46.2%) or CD59 (44.6%) expression associated with higher relapse rates (CD55: 33.3% vs. 8.6%, *p* = 0.013; CD59: 31.0% vs. 11.1%, *p* = 0.046) and shorter DFS (CD55: 56 vs. 70 months; CD59: 56 vs. 69 months). CD46 expression had no prognostic impact.	[20]
CD55	Before treatment	Clinical cohort; *n* = 480 unselected BC patients; no anti-HER2 therapy	Loss of CD55 associated with higher histological grade, poor prognostic indices, and shorter survival. Suggests complement-independent tumor-suppressive role of CD55 in untreated BC.	[49]
CD59	Before treatment	Clinical cohort; *n* = 520 unselected BC patients; no anti-HER2 therapy	Loss of CD59 correlated with poor survival. Suggests complement-independent role of CD59 in tumor differentiation in untreated BC.	[48]
CD59	Not specified	Clinical cohort + xenograft; *n* = 120 BC patients (±lung metastasis); MDA-MB-231 in vivo model	CD59 overexpression associated with worse prognosis. CD59 knockdown inhibited tumor cell growth both in vitro and in vivo.	[53]
Preclinical evidence				
CD55 CD59	On therapy (modelled)	Bioinformatics (KM Plotter) + in vivo shRNA; HER2+ BC preclinical models	CD55 and CD59 expression inversely correlated with relapse-free survival in HER2+ BC (KM Plotter). shRNA knockdown of CD55/CD59 in HER2+ tumors enhanced T + P therapeutic efficacy in vivo.	[15]
CD55 CD59	On therapy (modelled)	in vitro; SK-BR-3 and BT-474 (HER2-overexpressing BC cell lines)	Antibody-mediated blocking and shRNA knockdown of CD55/CD59 significantly enhanced trastuzumab-induced CDC. PI-PLC cleavage of GPI-anchored CD55/CD59 further potentiated complement-mediated lysis.	[19]
CD55 CD59 CD46	On therapy (modelled)	in vitro; HER2-overexpressing breast carcinoma (SK-BR-3, BT-474)	Trastuzumab deposited C1q to C9 but produced minimal lysis in human serum; combined neutralization of CD55, CD59 and CD46 raised trastuzumab-mediated lysis to 15%.	[55]
CD55	On therapy (modelled)	in vitro + clinical lymphoma cohort; SK-BR-3 (HER2+ BC) and primary NHL samples	CD55-directed siRNA restored trastuzumab-induced CDC (3% to 36% lysis). CD55 expression inversely correlated with CDC susceptibility in bulky lymphoma (r = −0.927).	[56]
CD55 CD59	On therapy (modelled)	in vitro; A549 and H157 (HER2+ NSCLC lines)	Neutralization of CD55 or CD59 increased herceptin-induced CDC (22% to 86% and 62%, respectively). Cisplatin downregulated CD55/CD59.	[47]
CD46 CD55 CD59	On therapy (modelled)	in vitro; BT-474, SK-BR-3 (BC), SKOV3 (ovarian), and Calu-3 (lung)	Lipoplex-delivered siRNAs targeting individual mCRPs enhanced CDC only with T + P combination. Simultaneous silencing of all three mCRPs increased CDC by 30% to 78%. mCRP knockdown also enhanced C3b opsonisation and macrophage-mediated CDCP.	[18]

Abbreviations: BC, breast cancer; CDC, complement-dependent cytotoxicity; CDCP, complement-dependent cellular phagocytosis; DFS, disease-free survival; GPI, glycosylphosphatidylinositol; mCRP, membrane-bound complement regulatory protein; NHL, non-Hodgkin lymphoma; NSCLC, non-small cell lung cancer; PI-PLC, phosphatidylinositol-specific phospholipase C; RFS, relapse-free survival; shRNA, short hairpin RNA; siRNA, small interfering RNA; T + P, trastuzumab + pertuzumab.

The same pattern extends beyond the membrane regulators. C8A, a component of the terminal complement pathway, inserts into the target membrane and binds to C9 to complete MAC formation. In the HERA trial, an analysis of 610 tumors revealed that patients with high C8A levels benefited from trastuzumab in terms of DFS, whereas no such benefit was observed in patients with low levels. Among patients who did not receive trastuzumab, the relationship was reversed, with high C8A levels associated with lower DFS (HR 3.27; *p* < 0.001) [57]. This suggests that C8A expression likely determines whether antibody-induced complement activation leads to lysis.

We found no study in which mCRP expression, C1q deposition or complement activation was measured in tumor material obtained after progression on anti-HER2 therapy (Table 1).

### 3.3. In Vitro Evidence: Neutralization of mCRP Restores CDC Sensitivity

The inability of trastuzumab alone to induce CDC against HER2+ tumor cells reflects not only the geometric problem of complement activation, but also the inhibition of the downstream complement cascade by mCRP.

Trastuzumab deposits complement components from C1q to C9 on HER2-overexpressing breast carcinoma cells, yet produces almost no lysis in human serum until CD55, CD59 and CD46 are neutralized [55], and blocking or knocking down the same regulators restores CDC in HER2-positive breast lines [19]. Treatment with phosphatidylinositol-specific phospholipase C (PI-PLC), which cleaves the GPI anchor of CD55 and CD59, further enhances complement-mediated lysis [19]. The same dependency is observed in HER2+ lung carcinoma cells, where the neutralization of CD55 or CD59 significantly enhances CDC [47]. In alignment with this finding, CD55-targeted siRNA increased trastuzumab-induced lysis in SK-BR-3 cells, and CD55 expression was inversely correlated with CDC susceptibility in primary lymphoma samples [56]. Indirectly supporting this, trastuzumab did not produce measurable CDC, and resistance to CDC induced by an EpCAM-specific antibody was most strongly associated with CD59 expression [34]. Mamidi et al. (2013) used chemically stabilized siRNAs delivered via lipoplex targeting all three mCRPs (CD46, CD55, and CD59) in HER2+ breast (BT-474, SK-BR-3), ovarian (SKOV3), and lung (Calu-3) cell lines. Individual suppression of mCRPs enhanced CDC only when trastuzumab and pertuzumab were combined. Simultaneous silencing of all three regulators had the strongest effect, increasing CDC by 30–78%, depending on the cell line [18] (Table 1). Silencing of mCRP also enhances C3b opsonization of tumor cells, enhancing macrophage-mediated phagocytic killing and linking complement evasion to resistance to both CDC and CDCP [18].

Tsao et al. (2022) also showed in vivo that shRNA-mediated suppression of CD55 and CD59 in HER2+ tumors enhanced the efficacy of the trastuzumab–pertuzumab combination in a mouse model [15] (Table 1).

Taken together, these findings indicate a role for mCRP, specifically CD55 and CD59 as mediators of resistance to anti-HER2 therapy: by shielding tumor cells from complement-mediated lysis, they limit the therapeutic effect of monoclonal antibodies.

## 4. C1q in the HER2+ Tumor Microenvironment: A Dual Role

C1q is the recognition molecule of the classical complement pathway, but accumulating evidence shows that it also exerts biological effects within the tumor microenvironment that are independent of complement cascade activation [27]. In cancer, C1q can act as either a tumor suppressor or a tumor promoter depending on the context.

### 4.1. Antitumoral Functions of C1q in HER2+ Disease

The most direct evidence for the tumor-suppressive role of C1q in HER2-driven carcinogenesis comes from Bandini et al. (2016), who compared mammary tumor progression in C1q-deficient (neuT-C1KO) and C1q-competent neuT transgenic mice, a model of HER2/neu-driven breast cancer [25]. C1q-deficient mice show significantly accelerated tumor incidence, along with earlier spontaneous lung metastases [25]. This effect was not due to impaired activation of the classical complement pathway, as C3 fragment deposition at the tumor site was not reduced in C1q-deficient mice. Instead, accelerated tumor growth is associated with reduced activation of the tumor suppressor WWOX (WW domain-containing oxidoreductase), higher expression of the Her2/neu oncoprotein on tumor cell membranes, and increased intratumoral vessel density [25].

These findings indicate that C1q exerts a direct, complement-independent antitumor effect in HER2+ mammary carcinogenesis by inducing apoptosis in tumor cells and inhibiting angiogenesis [25]. An earlier study by the same group showed that C3-deficient neuT mice also develop accelerated tumor progression [58], suggesting that the products of complement activation also contribute to tumor immune control.

Macagno et al. (2022) confirmed the same C1q–WWOX–HER2 axis in the same neuT model, using an anti-HER2 DNA vaccine, where C1q deposition was associated with WWOX activation and HER2 downregulation [59]. Here, tumor control was driven mainly by the direct and ADCC-mediated activity of antibodies, with CDC and CDCC playing only a minor role unlike the therapeutic trastuzumab–pertuzumab combination, in which classical pathway activation is a significant effector mechanism [15,18]. Together, these data indicate that complement proteins limit HER2-driven carcinogenesis in two ways: through activation of the complement cascade and through complement-independent mechanisms. These experimental observations are reinforced by two independent clinical and preclinical datasets: high *C1Q* gene expression is associated with better OS in HER2+ breast cancer [60] and intratumoral deposition of C1q is required for the therapeutic effect of the trastuzumab–pertuzumab combination in preclinical models [15].

The C1q-WWOX-dependent antitumor mechanism was first described in prostate cancer [61], and Kaur et al. (2016) showed that C1q induces apoptosis in ovarian cancer cells through the TNF-α pathway and mTOR inhibition [62]. This means that the tumor-suppressive functions of C1q are not unique to HER2-positive breast cancer.

### 4.2. Protumoral Functions of C1q

Multiple studies have shown that locally synthesized C1q in the tumor microenvironment can promote tumor progression, independent of the complement cascade. Bulla et al. (2016) found that C1q promotes adhesion, migration and proliferation of tumor cells in several human tumors, including breast cancer, and that in a mouse model of melanoma, C1q deficiency slowed tumor growth through reduced angiogenesis [63]. This protumoral pattern recurs through different mechanisms: in malignant pleural mesothelioma, C1q binds hyaluronic acid and drives adhesion and proliferation through ERK1/2, JNK and p38 signaling [64]; in gliomas, high C1q expression is associated with poor prognosis [65]; and in colorectal carcinoma, C1q-expressing macrophages engage cancer stem cells via the C1q receptor, triggering SETD8-mediated p53 inactivation and tumor progression [66]. In all the examples, C1q acts in the tumor’s favor, which stands in direct opposition to its suppressive role in HER2-positive disease.

In addition to C1q, other components of the C1 complex exhibit complement-independent functions in breast cancer. Minery et al. (2026) demonstrated that tumor cell-intrinsic C1r and C1s are preferentially expressed in triple-negative breast cancer (TNBC) cells, where they maintain cancer cell viability through an intracellular mechanism that is independent of extracellular complement activation [67]. Furthermore, TNBC cells expressing C1r and C1s secrete chemoattractant chemokines (CCL2, CCL20, CXCL1, CXCL2, and CXCL10) that attract myeloid cells but also cytotoxic T cells to the tumor microenvironment, and their expression correlates with improved overall survival [67]. These findings show that the components of the classical complement initiation complex modulate tumor biology through complement-independent pathways that diverge between breast cancer subtypes. C1q therefore plays a dual role that is dependent on the tumor type and microenvironment. In most solid tumors, locally produced C1q promotes tumor progression through complement-independent mechanisms. In HER2+ breast cancer, however, the balance is reversed. A predominantly protective role in this subtype is supported by these three observations: C1q deficiency accelerates tumor progression in vivo without any change in C3 fragment deposition [25]; *C1Q* expression correlates with favorable survival [60]; and C1q is required for the therapeutic efficacy of the trastuzumab–pertuzumab combination in preclinical models [15]. All three observations concern C1q inside the tumor, which is produced there rather than delivered from the blood. Therefore, measuring plasma C1q cannot be used to assess it. This places HER2-positive breast cancer in the category of complement-protective tumors [16,17]. In our opinion, the fact that the same molecule reverses direction between tumor types is the strongest argument for treating the role of complement as contingent rather than fixed.

## 5. Discussion

The evidence reviewed here defines HER2-positive breast cancer as a complement-protective tumor in the classification proposed by Roumenina et al. (2019) [16], a category in which complement activation limits malignant progression rather than accelerating it. In an attempt to find a place for this molecular subtype of breast cancer, we identified three independent lines of evidence regarding HER2-positive disease: (1) complement activation is an effector of the combination of trastuzumab and pertuzumab; (2) overexpression of mCRP in the tumor is associated with a worse outcome in patients treated with trastuzumab; and (3) C1q suppresses HER2-induced carcinogenesis in breast cancer through complement-independent mechanisms. The classification of HER2-positive breast cancer as a complement-protective tumor requires precision, as the three lines of evidence outlined above do not operate through a single mechanism. The first two of these relate to the complement cascade itself: its activation in the classical pathway by the trastuzumab–pertuzumab combination [15] and, conversely, the overexpression of membrane regulators that block it [20]. The third concerns C1q, acting as a molecule that limits HER2-induced carcinogenesis through the complement-independent WWOX axis [25], rather than triggering the classical complement cascade. The other subtypes of breast cancer have been studied in different terms, and the available data do not support the same conclusion [67,68].

What unites the evidence we have proposed for the affiliation of HER2-positive breast cancer to complement-protective tumors is not a shared mechanism, but a shared direction of action: each of these complement-related molecules acts against the tumor, not in its favor.

The distinctive feature of HER2-positive breast cancer is that therapeutic antibodies direct complement activity against the tumor, turning a system that in most other malignancies is tumor-promoting into a tumor-suppressive one. In this context, overexpression of mCRP in the tumor takes on a new significance: the same regulators that in an untreated tumor are not involved in the cascade, but upon therapy, block CDC and are associated with resistance.

The strongest support for our first line of evidence that HER2-positive breast cancer is a complement-protective tumor comes from Tsao et al. (2022), who demonstrated in preclinical models that the synergistic therapeutic effect of the combination of trastuzumab and pertuzumab depends on the classical activation of the complement pathway through CDC and CDCP [15]. This study repositions complement from a minor effector to an essential mechanism of action of the most widely used anti-HER2 combination regimen. Mamidi et al. (2013) confirmed that the trastuzumab–pertuzumab combination activates complement on HER2+ tumor cells (deposition of C3 fragments), although effective CDC is only achieved with simultaneous knockdown of mCRP CD46/CD55/CD59 (30–78% lysis) [18].

Overexpression of mCRP in HER2+ tumor cells, especially CD55 and CD59, is a clinically relevant mechanism of immune evasion and is associated with shorter disease-free survival (DFS) in patients treated with trastuzumab [20], identifying mCRP overexpression as a potentially modifiable determinant of treatment outcome.

If HER2-positive breast cancer is complement-protective, then novel interventions that enhance complement activation—biparatopic antibodies such as zanidatamab [22], complement-redirecting immunoconjugates [23,69], and Fc-engineering which promotes hexamerization [31]—work to the patient’s advantage, while overexpression of mCRP, which allows tumors to evade complement-mediated lysis, is itself a therapeutic target. Taken together, these approaches share a common rationale: each either provides the geometry that anti-HER2 antibodies lack or removes regulators that block the cascade downstream of C1q (Table 2).

Zanidatamab is a biparatopic anti-HER2 antibody that binds ECD2 and ECD4 in a trans configuration and induces a unique pattern of HER2 clustering on the tumor cell surface, resulting in potent CDC, a property not observed with trastuzumab or pertuzumab alone [22]. Zanidatamab received accelerated FDA approval for HER2+ biliary tract cancer in November 2024 [70]. It is currently being evaluated in a phase 3 trial (EmpowHER-303, NCT06435429) in HER2+ metastatic breast cancer [71]. Early phase data in first-line metastatic and neoadjuvant treatment demonstrate antitumor activity with a manageable safety profile [72,73]. If confirmed in Phase 3, zanidatamab would represent a new class of anti-HER2 therapy where complement activation is an important and targeted mechanism of action.

The idea that targeted complement activation can eliminate HER2+ tumor cells is further supported by experiments with FHR4-based immunoconjugates, which increase C3b/C5b-9 deposition and mediate CDC and CDCP on HER2+ cells in vitro [23], and with complement-activating multimeric immunotherapeutic complexes (CoMiX), which reduce tumor growth in xenograft models [69]. The different CoMiX constructs act via either the alternative or classical complement pathway, and their in vivo efficacy is lower than that of trastuzumab [69], indicating that complement activation is only part of the attack against HER2+ tumor cells.

IgG Fc engineering strategies, such as mutations that enhance hexamerization (e.g., E430G), increase the efficiency of C1q binding and potentiate CDC [31].

RNA interference strategies targeting mCRP have demonstrated proof of concept in vitro, although clinical translation will likely require tumor-selective delivery systems.

These emerging strategies are part of a broader effort to exploit complement as a therapeutic target in cancer immunotherapy, including both complement-enhancing and complement-inhibiting strategies, tailored to the specific tumor context [74]. However, they are at different stages of development, with zanidatamab being the most advanced in late-stage clinical trials, while the others are still preclinical.

These strategies also carry risks, and two of them deserve attention. The first arises from the lack of local targeting when complement regulation is manipulated. What the loss of mCRP costs is seen in paroxysmal nocturnal hemoglobinuria, where the absence of CD55 and CD59 from erythrocytes causes intravascular hemolysis and thrombosis [75]. To avoid the risk that silencing or neutralizing mCRP acts on the host rather than specifically on the tumor, bispecific antibodies have been developed that bind mCRP and a tumor antigen simultaneously; so far for targets other than HER2 [76]. The second risk concerns systemic infusion toxicity arising from local complement overactivation [77]. Rituximab, an anti-CD20 antibody that induces potent CDC, is an example. During the first infusion, complement activation precedes the release of TNF-α, IL-6 and IL-8, and the level of activation appears to be related to the severity of the side effects [78]. For such Fc constructs, and the way they are administered, a balance needs to be sought more actively between complement activations strong enough for tumor lysis and infusion toxicity.

The mechanistic case assembled here rests largely on cell lines and mouse models, and the clinical evidence is retrospective. Direct measurement of complement activation during anti-HER2 therapy has not been reported, and no clinical dataset distinguishes CDC from CDCP or from ADCC. These are the measurements the next studies should make.

This context-dependence also sets limits on how categorically HER2-positive breast cancer can be called complement-protective. First, the protective role of complement in HER2-positive breast cancer is not an intrinsic property of the tumor and becomes so in the presence of therapeutic antibodies that direct complement activity against the tumor. Membrane-bound regulators are associated with poor outcomes only in patients receiving anti-HER2 antibodies [20]; in untreated cohorts, their loss is associated with worse survival [48,49], although these cohorts are unselected rather than HER2-specific. Therefore, we can more accurately define HER2-positive breast cancer as conditionally complement-protective, distinguishing it from tumors in which complement favors progression. Second, the favorable prognostic association of C1q may not be the result of complement-mediated cytotoxicity alone, because C1q also suppresses HER2 signaling by activating WWOX, independent of the cascade [25]. Both mechanisms limit tumor growth, but current data are insufficient to indicate which is decisive for patients. The clinical evidence remains limited to retrospective cohorts, transcriptomic survival analyses, and one biomarker analysis in a randomized trial, and complement activation has not been measured directly during anti-HER2 therapy.

## 6. Conclusions

HER2-positive breast cancer is a conditionally complement-protective tumor in which antibody-directed complement activation contributes to therapeutic efficacy and overexpression of mCRP leads to resistance. The novel therapeutic strategies we presented, including biparatopic antibodies such as zanidatamab, complement-redirecting immunoconjugates, Fc-engineering, and mCRP-targeting agents, converge around a single principle: to enhance the complement cascade or to remove regulators that limit it. However, several questions remain that future studies need to clarify. What is the relative contribution of CDC, CDCP, and ADCC to the clinical efficacy of anti-HER2 antibodies? Most mechanistic evidence still comes from preclinical models. Can monitoring of complement biomarkers, such as intratumoral C1q deposition, circulating activation products, and mCRP levels, predict response to therapy? What determines the differential role of C1q in different tumors, and could this help to distinguish patients who would benefit from complement-enhancing therapy?

Two areas remain largely unexplored: (1) the role of complement in the spread of metastases, including epithelial–mesenchymal transition and the formation of a premetastatic niche, has not yet been studied in HER2+ breast cancer [79], and (2) combination strategies that combine mCRP-targeting agents with complement-activating antibodies are a promising but largely untested therapeutic approach.

The development of complement biomarkers to guide anti-HER2 therapy faces two major technical obstacles. There is no generally accepted threshold value for the immunohistochemical assessment of mCRP (Table 1), and complement activation products are generated ex vivo, necessitating blood collection in EDTA tubes and processing under controlled conditions to ensure data comparability across laboratories [80].

Complement plays a larger role in tumor biology than is often recognized, although the field has begun to change in recent years. A deeper understanding of complement biology in HER2-positive breast cancer, ranging from the mechanisms of antibody-induced complement activation to the strategies by which tumors resist and evade the immune response, could stimulate the development of more effective therapeutic approaches guided by complement biomarkers for this disease.

## Figures and Tables

**Table 2:** Emerging therapeutic strategies aimed at enhancing complement-mediated tumor killing in HER2-positive breast cancer.

Agent/ Approach	Complement-Enhancing Mechanism	Complement Pathway Targeted	Development Stage	References
Biparatopic antibody Zanidatamab	Binds ECD2 and ECD4 to adjacent HER2 molecules in a trans configuration for HER2 clustering and high surface Fc density—allows efficient C1q recruitment	Classical	FDA approval for HER2+ biliary tract cancer (2024); Phase 3 in HER2+ metastatic breast cancer (EmpowHER-303)	[22,70,71]
Complement-redirecting immunoconjugate FHR4-based CoMiX	Delivers FHR4 to the tumor cell surface for competition with Factor H—prevents alternative pathway regulation; C3b deposition—CDC and CDCP	Alternative	Preclinical (in vitro and xenograft models)	[23,69]
Fc engineering Hexamerisation- enhancing mutations (e.g., E430G)	Promotes noncovalent Fc:Fc interactions and assembly of hexameric IgG platforms on the cell surface—increases C1q binding avidity	Classical	Preclinical (platform technology)	[31]
mCRP silencing Lipoplex-delivered siRNA/shRNA against CD46, CD55, CD59	Removes mCRP and restores convertase activity and MAC assembly on tumor cells—enhances both CDC and CDCP	Classical (regulation)	Preclinical; clinical translation requires tumor-selective delivery	[18,19]

Abbreviations: CDC, complement-dependent cytotoxicity; CDCP, complement-dependent cellular phagocytosis; CoMiX, complement-activating multimeric immunotherapeutic complexes; ECD, extracellular domain; FDA, U.S. Food and Drug Administration; FHR4, factor H-related protein 4; MAC, membrane attack complex; mCRP, membrane-bound complement regulatory protein; shRNA, short hairpin RNA; siRNA, small interfering RNA.

## Data Availability

No new data were created or analyzed in this study. Data sharing is not applicable to this article.

## Abbreviations

The following abbreviations are used in this manuscript:

ADCC	Antibody-dependent cellular cytotoxicity
ADCP	Antibody-dependent cellular phagocytosis
CDC	Complement-dependent cytotoxicity
CDCC	Complement-dependent cellular cytotoxicity
CDCP	Complement-dependent cellular phagocytosis
CoMiX	Complement-activating multimeric immunotherapeutic complexes
DFS	Disease-free survival
EGFR	Epidermal growth factor receptor
*ERBB2*	Erb-B2 receptor tyrosine kinase 2 (gene encoding HER2)
HER2	Human epidermal growth factor receptor 2
MAC	Membrane attack complex
mCRP	Membrane-bound complement regulatory proteins
mTOR	Mammalian target of rapamycin
NK cells	Natural killer cells
OS	Overall survival
RFS	Relapse-free survival
SCID	Severe combined immunodeficiency
shRNA	Short hairpin RNA
siRNA	Small interfering RNA
TNBC	Triple-negative breast cancer
TNF-α	Tumor necrosis factor alpha
WWOX	WW domain-containing oxidoreductase
